# Noninvasive detection of pancreatic ductal adenocarcinoma using the methylation signature of circulating tumour DNA

**DOI:** 10.1186/s12916-022-02647-z

**Published:** 2022-11-25

**Authors:** Huanwen Wu, Shiwei Guo, Xiaoding Liu, Yatong Li, Zhixi Su, Qiye He, Xiaoqian Liu, Zhiwen Zhang, Lianyuan Yu, Xiaohan Shi, Suizhi Gao, Huan Wang, Yaqi Pan, Chengcheng Ma, Rui Liu, Menghua Dai, Gang Jin, Zhiyong Liang

**Affiliations:** 1 Department of Pathology, Molecular Pathology Research Center, Peking Union Medical College Hospital, Chinese Academy of Medical Sciences & Peking Union Medical College, No.1, Shuaifuyuan Wangfujing, Dongcheng District, Beijing, 100730 China; 2 Department of Hepatobiliary Pancreatic Surgery, Changhai Hospital, Navy Medical, University (the Second Military Medical University), No.168, Changhai Road, Shanghai, 200433 China; 3 Department of General Surgery, Peking Union Medical College Hospital, Chinese Academy of Medical Sciences & Peking Union Medical College, No.1, Shuaifuyuan Wangfujing, Dongcheng District, Beijing, 100730 China; 4 Singlera Genomics (Shanghai) Ltd., No. 500, Furonghua Road, Shanghai, 201203 China

**Keywords:** PDAC, Early detection, Circulating tumour DNA methylation, Methylation haplotype blocks

## Abstract

**Background:**

Pancreatic ductal adenocarcinoma (PDAC) has the lowest overall survival rate primarily due to the late onset of symptoms and rapid progression. Reliable and accurate tests for early detection are lacking. We aimed to develop a noninvasive test for early PDAC detection by capturing the circulating tumour DNA (ctDNA) methylation signature in blood.

**Methods:**

Genome-wide methylation profiles were generated from PDAC and nonmalignant tissues and plasma. Methylation haplotype blocks (MHBs) were examined to discover de novo PDAC markers. They were combined with multiple cancer markers and screened for PDAC classification accuracy. The most accurate markers were used to develop PDACatch, a targeted methylation sequencing assay. PDACatch was applied to additional PDAC and healthy plasma cohorts to train, validate and independently test a PDAC-discriminating classifier. Finally, the classifier was compared and integrated with carbohydrate antigen 19-9 (CA19-9) to evaluate and maximize its accuracy and utility.

**Results:**

In total, 90 tissues and 546 plasma samples were collected from 232 PDAC patients, 25 chronic pancreatitis (CP) patients and 323 healthy controls. Among 223 PDAC cases with known stage information, 43/119/38/23 cases were of Stage I/II/III/IV. A total of 171 de novo PDAC-specific markers and 595 multicancer markers were screened for PDAC classification accuracy. The top 185 markers were included in PDACatch, from which a 56-marker classifier for PDAC plasma was trained, validated and independently tested. It achieved an area under the curve (AUC) of 0.91 in both the validation (31 PDAC, 26 healthy; sensitivity = 84%, specificity = 89%) and independent tests (74 PDAC, 65 healthy; sensitivity = 82%, specificity = 88%). Importantly, the PDACatch classifier detected CA19-9-negative PDAC plasma at sensitivities of 75 and 100% during the validation and independent tests, respectively. It was more sensitive than CA19-9 in detecting Stage I (sensitivity = 80 and 68%, respectively) and early-stage (Stage I-IIa) PDAC (sensitivity = 76 and 70%, respectively). A combinatorial classifier integrating PDACatch and CA19-9 outperformed (AUC=0.94) either PDACatch (0.91) or CA19-9 (0.89) alone (*p* < 0.001).

**Conclusions:**

The PDACatch assay demonstrated high sensitivity for early PDAC plasma, providing potential utility for noninvasive detection of early PDAC and indicating the effectiveness of methylation haplotype analyses in discovering robust cancer markers.

**Graphic Abstract:**

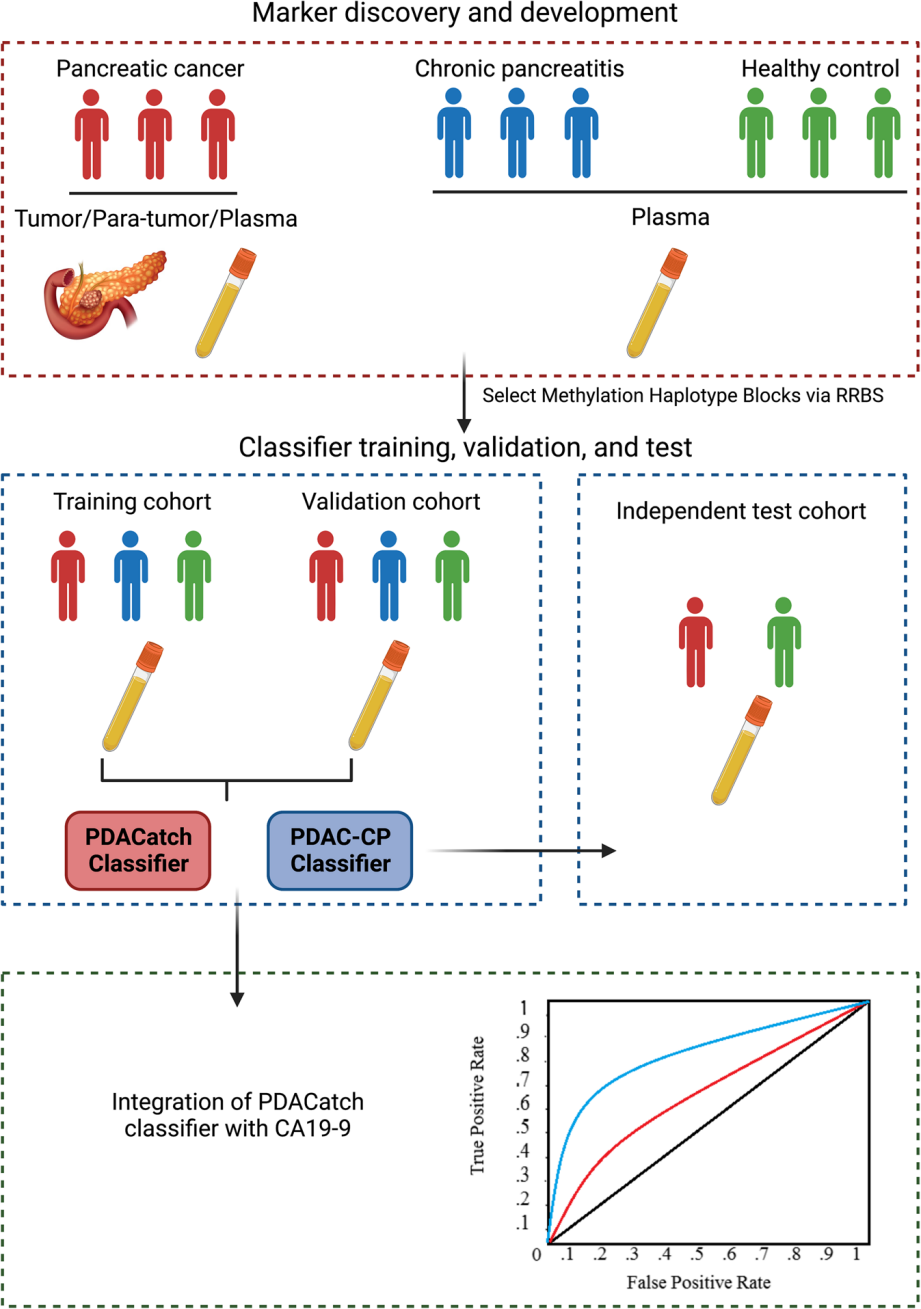

**Supplementary Information:**

The online version contains supplementary material available at 10.1186/s12916-022-02647-z.

## Background

Pancreatic ductal adenocarcinoma (PDAC) is widely considered one of the most lethal diseases worldwide. One main reason for its high mortality rate is the lack of effective early detection methods. Early symptoms, which typically include abdominal and back pain, diarrhea, weight loss and jaundice, are nonspecific for PDAC and may be associated with other gastrointestinal diseases. This complication is particularly prominent in the differential diagnosis between chronic pancreatitis (CP) and PDAC [1].

Currently, carbohydrate antigen 19-9 (CA19-9) is the most widely used clinical serum marker to detect PDAC, and it can reach a sensitivity and specificity of 75–90% in symptomatic patients prior to resection [2, 3]. However, the low sensitivity in early-stage disease has limited its use in early detection protocols. Its practical application in early cancer detection is also hampered by false negatives in Lewis-negative individuals (5–10% of the general population) [4]. Moreover, several large-population studies have demonstrated that CA19-9 is ineffective in detecting PDAC in asymptomatic populations due to its high false-positive rate in conditions of inflammation and nonpancreatic cancers [5, 6]. Endoscopic ultrasound-guided fine needle aspiration (EUS-FNA) is a commonly used method to obtain pathological diagnosis; however, it is invasive and has been linked to bleeding and/or tumour dissemination [7]. Therefore, noninvasive and more accurate methods to detect early PDAC are highly desirable for improving the clinical outcomes of PDAC patients.

In recent years, aberrant DNA methylation has been proposed as a promising marker for noninvasive cancer detection [8]. DNA methylation patterns are profoundly altered in the genome of malignant cells during tumourigenesis and progression [9]. The epigenetic signatures of cancer cells can be utilized to detect circulating tumour DNA (ctDNA) molecules in circulating cell-free DNA (cfDNA) samples [10], which have been explored to develop blood markers for the early screening of PDAC [11–17].

To further improve the accuracy and robustness of ctDNA methylation markers for early PDAC screening from previous studies, we first employed novel methylation haplotype blocks (MHBs) for marker discovery and validation. MHBs are discrete genomic regions that have tightly coupled CpG methylation sites (i.e., methylation haplotypes) [18] that have been shown to have superiority over methylation on individual CpG sites and were screened as PDAC markers in prior studies, with respect to both sensitivity and specificity in deconvoluting trace amounts of ctDNA from total cfDNA [19]. While MHB analyses have been utilized to identify markers for noninvasive cancer screening in several cancer types [18, 19], we were the first to apply it for PDAC marker screening and testing.

Second, we systematically developed new metrics to comprehensively interrogate methylation haplotypes as well as unmethylation haplotypes within MHBs (see Additional file 1: Supplementary Methods [19–28] for details) to enlarge the pool of potential PDAC markers. These de novo markers were combined with literature-based candidate markers to form a highly informative marker panel, which was then developed into PDACatch, an ultrasensitive targeted methylation sequencing assay for detecting ctDNA methylation signatures in blood. The PDACatch classifier was built and validated in PDAC and control plasma samples and was tested in a single-blinded manner to confirm its accuracy. It was further compared with CA19-9 in the ability to discriminate PDAC plasma from healthy controls. Our results showed that the PDACatch classifier was more sensitive than CA19-9 in detecting plasma of early-stage PDAC, suggesting its potential to be optimized into diagnostics to detect PDAC early in blood.

## Methods

### Participants

**Fig. 1:**
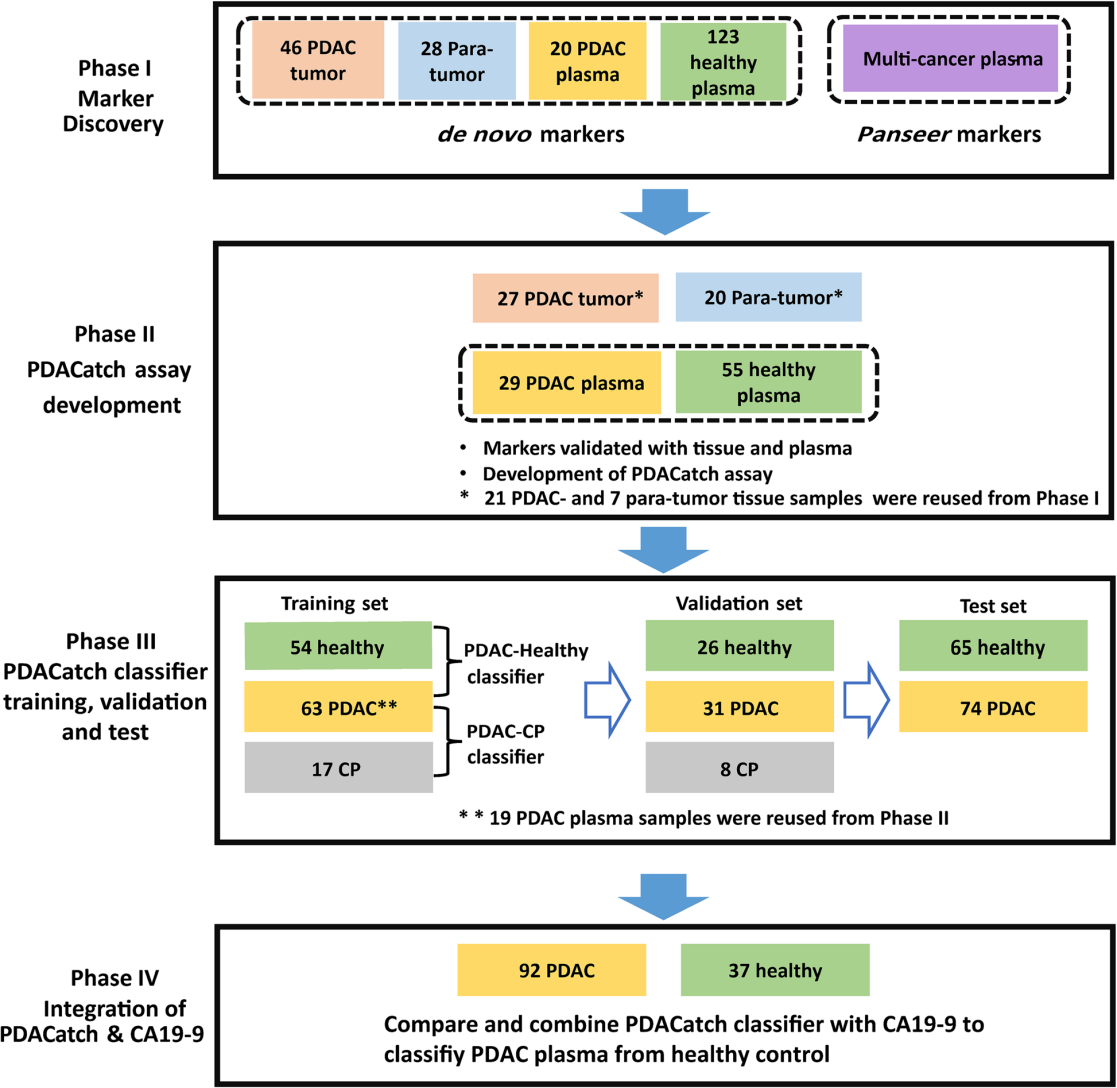
Study flow diagram of four sequential case–control studies for marker discovery and validation

All PDAC plasma samples except the test cohort in Phase III (Fig. 1) were collected prior to surgery (pancreaticoduodenectomy or distal pancreatectomy) at the Peking Union Medical College Hospital (PUMCH) and Changhai Hospital, Navy Medical University (CHNMU) from Oct. 2017 to Oct. 2019. All PDAC tissues and matched adjacent nontumour tissues were obtained from surgical resection. All PDAC patients were pathologically diagnosed using specimens obtained from surgical resection. The slides were reviewed by two experienced pathologists (H.W. and Z.L.), and the diagnosis was confirmed. CP plasma samples were collected from CHNMU from May 2019 to Sept. 2019. The inclusion criteria for CP patients followed the international consensus [29]. Plasma samples of healthy individuals were collected from PUMHC and CHNMU from May 2018 to Sept. 2019. All cohorts provided informed consent. Samples of the test cohort in Phase III were purchased from ProteoGenex (Inglewood, CA, USA). A few tissue and plasma samples were used in two phases (Additional file 2: Fig. S1). This project was approved by the PUMHC Ethics Committee (No. JS-1490) and CHNMU Ethics Committee (No. CHEC2020-113).

PDAC patients were staged according to the eighth edition of the American Joint Committee on Cancer TNM Staging System. We defined early PDAC as cases with TNM Stage IA, IB, or IIA, which refer to patients without lymph node involvement or distant metastasis [30, 31].

### Plasma sample preparation, DNA extraction, RRBS and targeted methylation sequencing

All blood samples were collected in cfDNA BCT tubes (Streck). Plasma separation and plasma DNA extraction were performed as previously described (23). Briefly, blood samples were centrifuged at 1600×*g* for 10 min at 4 °C. The supernatant was transferred and centrifuged again at 16,000×*g* for 10 min at 4 °C, and plasma was collected and aliquoted into nuclease-free tubes at −80 °C. Circulating cfDNA was extracted from plasma using a QIAamp Circulating Nucleic Acid Kit (Qiagen, 55114) according to the manufacturer’s instructions. Tissue samples were freshly obtained after surgical resection. Genomic DNA was extracted using a DNeasy Blood and Tissue Kit (Qiagen).

Reduced representation bisulfite sequencing (RRBS) or targeted methylation sequencing was performed as previously described [19, 32]. For each sample, 50–100 ng of genomic DNA or 20 ng of plasma DNA was used. All libraries were paired-end sequenced on Illumina platforms for 150 cycles.

### Methylation haplotype measurements

Candidate methylation haplotype blocks (MHBs) were constructed as described previously [19]. The CpG sites within each MHB tend to be tightly coregulated on the epigenetic status at the level of single DNA molecules. We evaluated multiple block-level quantitative metrics to identify the most informative measurement for each target region. Such metrics included AMF (average methylation fraction), MHL (methylation haplotype load), UMHL (unmethylation haplotype load), MHFm (fully methylated haplotype fraction) and MHFu (fully unmethylated haplotype fraction).

#### AMF

AMF was defined as the average methylation level for all CpG sites in a specific target region. All detected CpG alleles were divided by all methylated CpG alleles of the target region

$$\frac{\sum_i^M{N}_{C,i}}{\sum_i^M\left({N}_{C,i}+{N}_{T,i}\right)}$$

where *i* represents a CpG site in this target region, *M* is the total number of CpG sites in this target region, *N*_*T,i*_ represents the number of thymines observed at CpG site *i* and *N*_*C*,*I*_ represents the number of cytosines observed at CpG site *i*.

#### MHL, MHL3, UMHL and UMHL3

MHL was defined as in Guo et al. [19], which is the normalized fraction of methylated haplotypes at different lengths.

$$\textrm{MHL}=\frac{\sum_{i=1}^l{w}_i\times P\left({MH}_i\right)}{\sum_{i=1}^l{w}_i}$$

where *l* is the length of haplotypes and *P*(*MH*_*i*_) is the fraction of fully successive methylated CpGs within *i* loci. *w*_*i*_ is the weight for the *i*-locus haplotype. The options for weights are *w*_*i*_ = *i* for MHL and *w*_*i*_ = *i*^3^ for MHL3. Similar to MHL and MHL3, UMHL and UMHL3 are the normalized fractions of unmethylated haplotypes at different lengths.

#### MHFm and MHFu

TheMHFm metric was computed for each fully methylated haplotype over each targeted region using the equation:

$${MHFm}_{i,h}=\frac{N_{i,h}}{N_i}$$

where *i* is the current locus, *h* is the current haplotype, *N*_*i,h*_ is the number of reads at the current locus containing the current haplotype, and *Ni* is the total number of reads covering the current locus. MHFu is the fraction of fully unmethylated haplotypes.

### Discovery of de novo markers

Target MHBs of the Phase II panel were selected from multiple sources: PDAC tissues vs. healthy plasma, PDAC tissues vs. para-tumour tissues, PDAC plasma vs. healthy plasma and literature searches.

When comparing the methylation profiles of PDAC tissues vs. healthy plasma and PDAC tissues vs. para-tumour tissues, the analytical process includes marker filtering and differentially methylated MHB selection. In a library, MHBs with a sequencing depth < 10 were set to NA. MHBs with an NA rate greater than 10% in all libraries were removed from the analyses. Furthermore, MHBs with variations less than 0.02 were removed. After filtering, MHBs with FDR-adjusted *p* values < 0.05 were selected.

In PDAC plasma vs. healthy plasma analysis, 20 healthy plasma samples were randomly selected to balance cases and controls, which was repeated 500 times. In each iteration, the selected 40 samples were randomly split into a marker discovery set (15 PDAC, 15 healthy) and a validation set (5 PDAC, 5 healthy). Differentially methylated MHBs were identified in the discovery set with the Wilcoxon rank sum test FDR < 0.05. A random forest model was built with these markers in the discovery set and validated in the validation set. If AUC ≥ 0.75, the identified MHBs were kept. MHBs identified over 300 times were selected for downstream analysis.

### PDAC classifier construction

#### Regional measurement selection

Methylation markers with low sequencing depth were filtered out. The remaining methylation markers detected in ≥ 90% of the training samples were kept for downstream analyses.

First, we determined the most discriminative measurements for each marker. For every measurement, a logistic regression model was built with one measurement at a time for the training samples using the Python package statsmodels (0.11.1) as follows:

$${h}_{\theta }\ (x)=g\left({\theta}^Tx\right)=\frac{1}{1+{e}^{-\left({\theta}_0+{\theta}_1x\right)}}$$

*h*_*θ*_(*x*): phenotype;*x*: measurement; *θ*_0_: intercept; *θ*_1_: coefficient of the measurement.

The *p* value of *θ*_1_ was returned when the model was built. Measurements of each individual marker were ranked based on the *p* values, and the measurement with the smallest *p* value was selected as a regional measurement to represent the methylation status of the marker.

#### Incremental feature selection and classifier construction

The number of markers was determined by incremental marker selection. After regional measurements of each target were selected, missing measurement values were imputed with the values of 5 nearest neighbours (KNN). The selected values of a marker were then scaled based on the median value and the 25–75% interquartile range. Then, the training samples were randomly split into 10 fractions: a support vector machine (SVM) model for each marker was built using 9 fractions and tested by the remaining 1 fraction. This process was repeated 10 times, during which the area under the curve (AUC) was calculated for each test and averaged. Note that we started with the marker with the smallest *p* value.

For a new marker, if its average AUC of the 10 tests did not decrease below the average AUC of the previously tested marker(s), this marker was included for classifier training. After all markers were tested, an SVM classifier for PDAC plasma was built using all included markers by classifying all training samples and was validated using the validation samples (Additional file 2: Fig. S2). Plasma samples with CA19-9 level information were selected to build a combinatorial classifier integrating the methylation classifier and serum CA19-9 level.

#### Statistical analyses

Statistical analyses were performed in R 3.5.0. In Phase II, chi-square tests were utilized to test the difference in methylation level distribution between the case and control groups for each marker: values of each marker were assigned to 10 windows evenly distributed from 0 to 1 for the PDAC and healthy groups. A chi-square contingency test was performed to test whether the distribution of samples in each window was identical between the PDAC and healthy groups. Measurements with the smallest chi-squared test *p* value of each target were selected as the methylation status of the corresponding target regions. Binomial confidence intervals for sensitivity and specificity were calculated using the Clopper-Pearson method. To assess whether the difference observed between AUCs from the combinatorial model (i.e., CA19-9+PDACatch) and the CA19-9 alone model was statistically significantly different from 0, we considered a test statistic *T* [(*T* = AUCCA19-9 − AUCCA19-9+PDACatch)2/(s2 CA19-9 + s2 PDACatch)] [10], which looks at the difference in AUC between the two models divided by the sum of the variances from the two models. The fact that this test statistic followed a *χ*^2^ distribution with 1 degree of freedom under the null hypothesis was used to calculate a resulting *p* value. A bootstrap percentile confidence interval (CI) approach was used to estimate a 95% CI for the AUC (1000 times).

## Results

### Study design and sample description

This study utilized a total of 90 tissues (52 PDAC tumours, 38 matched para-tumour tissues) and 546 plasma samples (198 PDAC, 25 CP and 323 healthy controls) to sequentially develop a PDACatch assay (Fig. 1, Additional file 2: Fig. S1, Tables 1 and 2). PDAC samples were collected from 232 PDAC patients (18 PDAC patients provided both tissue and plasma samples). Among 223 PDAC patients with known stage information, 43/119/38/23 cases were Stage I/II/III/IV (Additional file 2: Fig. S2A). In Phase I, we discovered de novo PDAC-specific markers by analysing the genomic DNA methylation profiles of PDAC tumours, normal tissues and plasma samples using the RRBS method [19]. In Phase II, markers were tested in additional tissue and plasma samples for their PDAC-discriminating accuracy. The most informative markers were selected to develop a targeted sequencing assay, PDACatch. In Phase III, PDAC classifiers were built and validated in 199 plasma samples to separate PDAC patients from healthy individuals or CP patients. Furthermore, we conducted a single-blinded test of the PDACatch classifier using an independent cohort of PDAC plasma and healthy controls. In Phase IV, we compared PDACatch and CA19-9’s performances in classifying PDAC plasma and explored an integrated classifier to further improve the accuracy. Note that a small number of tissue and plasma samples were shared in multiple phases of this study (see details in the relevant “Results” sections).

**Table 1:** Demographic and clinicopathological features of the study cohorts

Phase	I—Marker discovery				II—Assay development				III—Training and validation			III—Test	
Pathology	Tissue		Plasma		Tissue		Plasma		Plasma			Plasma	
	PDAC	Para-tumour	PDAC	Healthy	PDAC	Para-tumour	PDAC	Healthy	PDAC	CP	Healthy	PDAC	Healthy
**Sample size**	46	28	20	123	27	17	29	55	94	25	80	74	65
**Age (years)**													
**Median**	62	61	62	54	63	54	59	53	63	55	55	63	64
***p*value**	0.1468*		<0.0001*		0.0462*		0.0042*		<0.0001**			0.2721*	
**Range**	41–77	30–76	41–76	23–90	41–83	36–83	35–77	17–76	35–80	25–68	18–79	44–86	49–80
**Gender: Male/female**	23/23	10/18	12/8	49/74	15/12	9/8	11/16	22/32	56/38	19/6	35/45	30/44	19/46
**Clinical stage**													
**I**	14	-	7	-	10	-	6	-	23	-	-	3	-
**II**	15	-	7	-	9	-	14	-	47	-	-	51	-
**III/IV**	16	-	4	-	7	-	4	-	23	-	-	20	-
**N/A**	1	-	2	-	1	-	5	-	1	-	-	0	-
**Tumour size (cm)**													
**Median**	3.4	2.5	2.8	-	2.9	2.7	3.3	-	3	-	-	-	-
**Range**	1.5–8.0	1.5–3.5	1.5–8	-	1.8–8.0	1.0–7.5	1.6–9.0	-	1.0–8.0	-	-	-	-
**CA19-9 (U/ml)**													
**Median**	271.5	222	-	-	196.6	390.4	360.1	-	421.6	109.6	43	293	-
**Range**	0–3447	9–1514	-	-	0.6–1514	0.5–3447	2–1200	-	2–1200	1–1200	1–890	2–3386.5	-
**>37**	26	6	-	-	11	7	21	-	50	2	4	15	-

*PDAC* pancreatic ductal adenocarcinoma, *CP* chronic pancreatitis, *CA19-9* carbohydrate antigen 19-9

*Two-sided Mann-Whitney test

**Two-sided Kruskal-Wallis test

**Table 2:** Demographic and clinicopathological features of the study cohorts in Phase IV

Pathology	Plasma	
	PDAC	Healthy
**Sample size**	92	37
**Age (years)**		
**Median**	63.1	51.1
**Range**	35–80	18–79
**Sex: male/female**	56/36	15/22
**Stage**		
**I**	25	-
**II**	44	-
**III/IV**	23	-
**N/A**	0	-
**Tumour size (cm)**		
**Median**	3.0	-
**Range**	1–8	-
**CA19-9 (U/ml)**		
**Median**	471.5	4.2
**Range**	21.13–1200	1–9.57
**>37**	15	0

### Discovery of PDAC-specific methylation markers in tissue and plasma

**Fig. 2:**
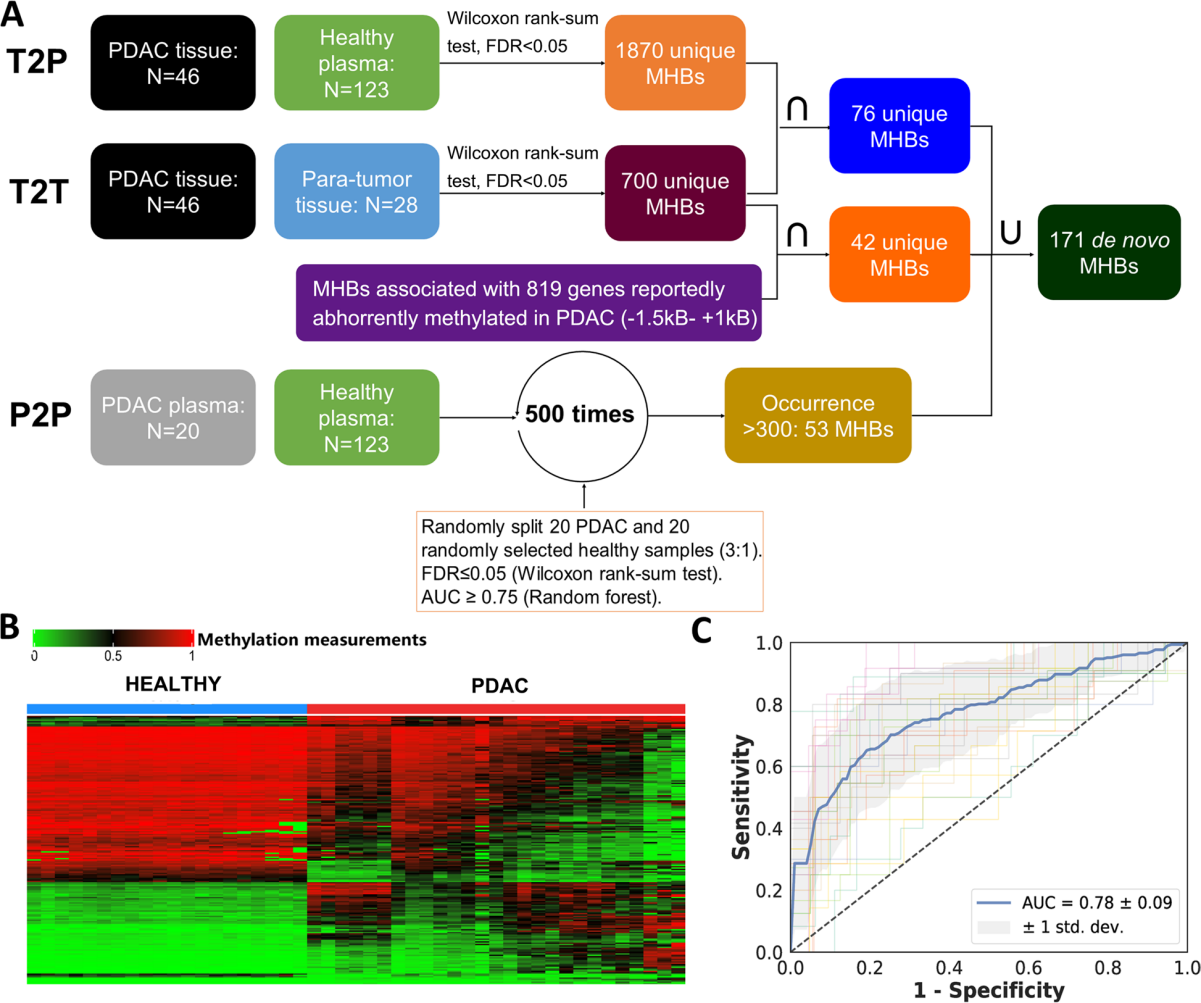
**A** PDAC-specific markers were discovered by T2T, T2P and P2P comparisons separately and then intersected and combined, as depicted in the figure. **B** Unsupervised hierarchical clustering of PDAC and para-tumour tissues based on their methylation measurements of the 750 assembled markers, which were ordered along the *Y* axis of the heatmap. **C** The receiver operating characteristic (ROC) curve of an SVM model built by using the 200 most discriminatory markers to cross-validate Phase II plasma samples

We searched predefined methylation haplotype blocks (MHBs) for de novo PDAC-specific DNA methylation markers by first profiling the methylation patterns of 46 PDAC tumours, 28 para-tumour tissues and 143 plasma samples (20 PDAC, 123 healthy) using RRBS (Table 1). Multiple MHB-specific metrics (see the “Methylation haplotype measurements” section for details) were used to quantify methylation levels to identify MHBs containing PDAC-specific methylation haplotypes as markers via PDAC tissue group vs. para-tumour tissue group (T2T), PDAC tissue vs. healthy plasma (T2P) and PDAC plasma vs. healthy plasma (P2P) comparisons (Fig. 2A). The first set of 76 markers was yielded by intersecting the 1870 T2P markers and 700 T2T markers (Wilcoxon rank sum test, Benjamini and Hochberg FDR <0.05). The second set comprised 42 T2T markers located within −1500 to +1000 bp of the transcription start sites of 819 genes exhibiting aberrant methylation changes in PDAC tissues or plasma [21–24]. The third set of 53 markers was selected from P2P markers via a model-based cross-validation marker selection process using an AUC value of over 0.75 as the cut-off for qualified markers. In total, 171 de novo markers were compiled for downstream analysis.

**Fig. 3:**
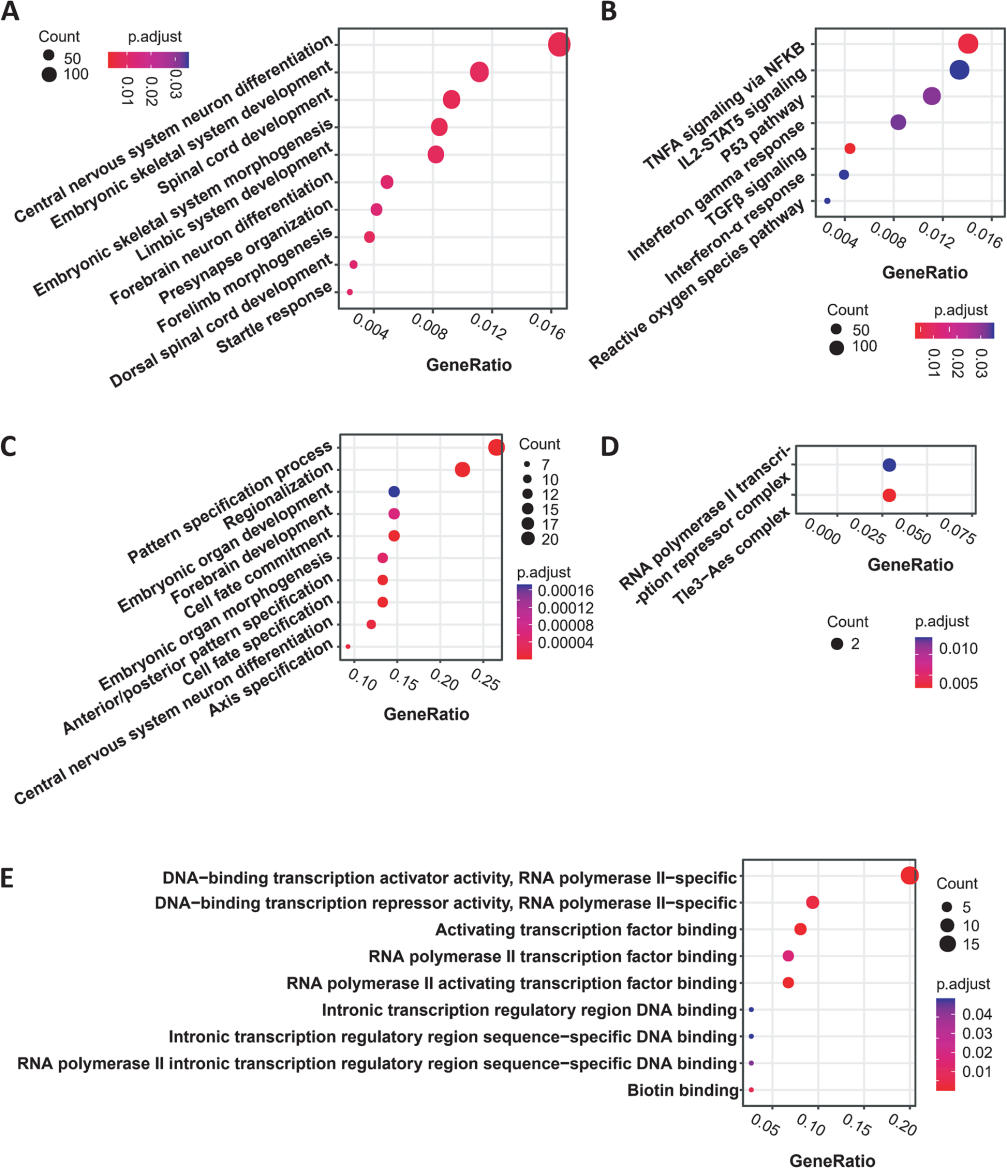
**A**, **B** Top 10 GO biological processes (**A**) and the 7 MSigDB hallmark cancer-related pathways (**B**) identified by GSEA that were enriched in the genes associated with the 171 de novo PDAC-specific markers. **C**–**E** Significantly enriched functional categories of genes associated with the 56 markers in the final PDACatch classifier, as identified by GO analysis. **C** Biological processes (top 20). **D** Cellular component. **E** Molecular function

Gene set enrichment analysis (GSEA) of these de novo MHBs revealed that multiple cancer-related pathways or biological processes were enriched in their associated genes (FDR < 0.05, Fig. 3A, B and Additional file 3: Table S1) [33], including 7 MsigDB hallmark pathways known to be dysregulated in PDAC (Fig. 3B) [23, 34]. Furthermore, the PDAC marker genes previously published in the literature were also highly enriched in our top marker-associated genes (hypergeometric test, Additional file 4: Table S2) [21–24]. These results strongly supported that the de novo markers we selected are involved in PDAC carcinogenesis.

### Marker optimization and PDACatch assay development

In Phase II, we further reduced the number of PDAC markers to minimize model overfitting caused by the imbalance of hundreds of features and limited samples. To this end, all the selected markers were integrated with the *PanSeer* assay [32], and their separation power was validated. *PanSeer* is a targeted methylation sequencing assay that is highly sensitive for detecting early-stage cancer signals in blood. It is also readily customized for different sets of targets, making it versatile for investigating different types of cancers.

A combination of the PDAC de novo markers with the *PanSeer* markers formed a starting set of 750 markers for further testing (Fig. 2B). We filtered them by using them to discriminate PDAC tumours from para-tumour tissues (*N* = 27 and 17, respectively). Among them, 21 PDAC and 7 para-tumour tissues were previously used in Phase I. They were reused in Phase II to confirm that RRBS-discovered markers can also be consistently detected by targeted methylation sequencing. The top 200 most discriminating markers (*p* < 0.05, Wilcoxon rank sum test) were selected and preliminarily filtered based on their ability to classify PDAC and healthy plasma (*N* = 29 and 55, respectively) via cross-validation (Fig. 2C) and the distribution of their methylation haplotype measurements in these samples (chi-square test, *p* < 0.01). The 185 most significant markers were chosen to develop the final version of the PDACatch assay to detect the PDAC marker signature in blood.

### Model building and evaluation of the PDACatch classifier for early PDAC detection

**Fig. 4:**
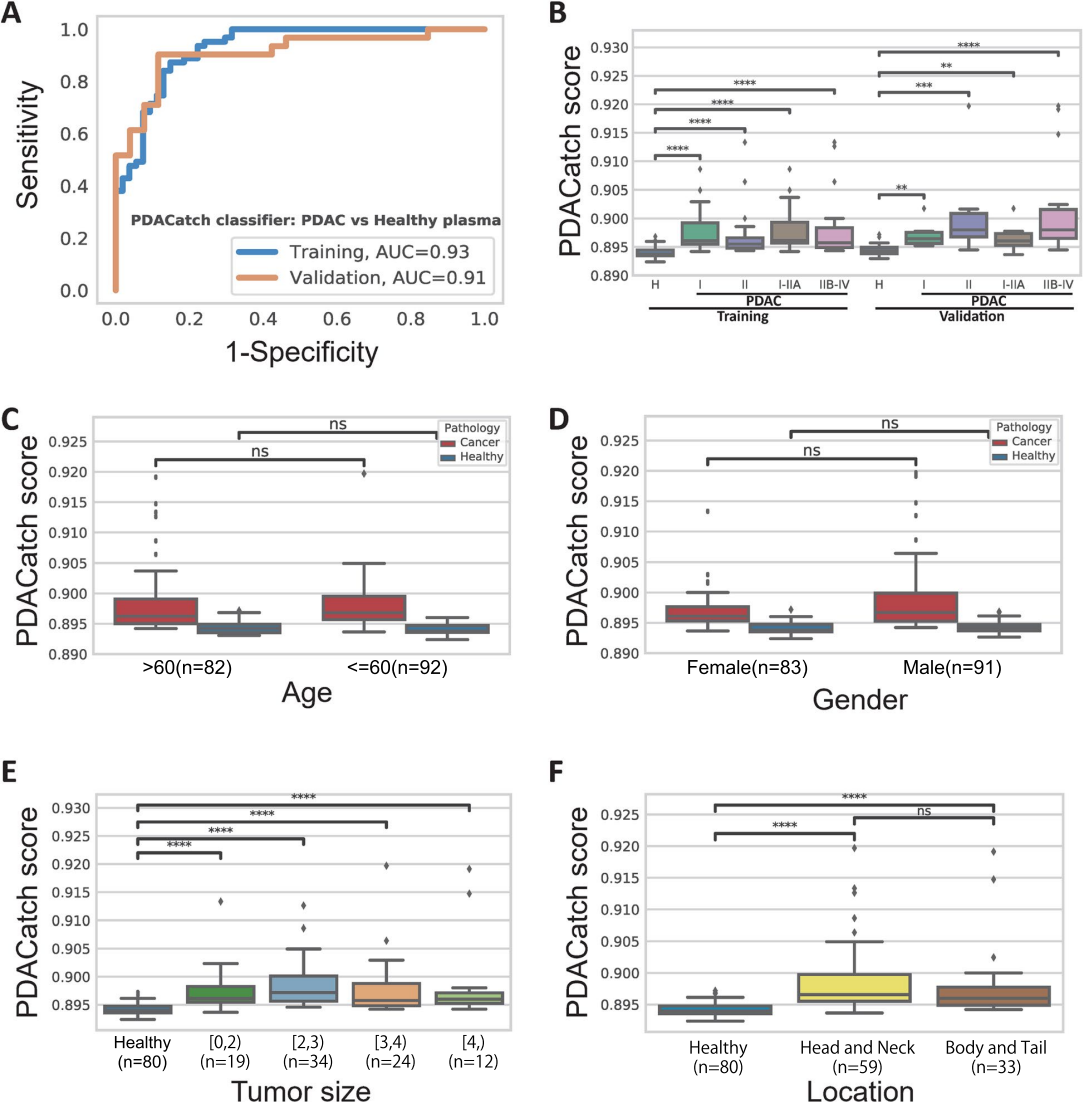
Performance of the PDACatch classifier in differentiating PDAC from healthy plasma and covariate analysis of the PDACatch classifier. **A** ROC curve of the PDACatch classifier distinguishing PDAC and healthy plasma in the training and validation cohorts. **B** The PDACatch classifier scores across different types of samples. In Panel **B**, samples were labelled with cohorts (Training/Validation), pathological types (H: Healthy; CP: chronic pancreatitis) and stages (I, II, I-IIA and IIB-IV). During covariate analysis, PDACatch scores of PDAC and healthy plasma samples were grouped by sex (**C**), age (**D**), tumour size (**E**) and tumour location (**F**). In **E**, brackets and parentheses indicate inclusion and exclusion of size, respectively. Wilcoxon rank sum test: ns, not significant (0.05 < *p* ≤ 1.0); *: 0.01 < *p* ≤ 0.5; **: 0.001e−03 < *p* ≤ 0.01; ***: 0.0001 < *p* ≤ 0.001; ****: *p* ≤ 0.0001

We then sought to develop a PDAC early detection classifier to separate PDAC plasma from healthy controls by the PDACatch assay. To this end, 94 PDAC and 80 healthy samples, which were age- and sex-matched, were randomly split into a training set and a validation set at a 2:1 ratio (Fig. 1 and Table 1). The training set included 19 PDAC plasma samples that were previously tested in Phase II and had sufficient remaining cfDNA. This was to increase the size of the training set to improve the trained classifier’s robustness; however, no samples were reused in validation to prevent biasing the validation results. Using training samples, the 56 most discriminatory markers for PDAC were identified by 10-fold cross-validation incremental feature selection (Additional file 2: Fig. S3 and Additional file 5: Table S3) to build an SVM-based classifier with a high AUC of 0.93 in the training set (sensitivity = 71%, specificity = 91%) (Fig. 4A). The PDACatch classifier was then validated in the left-out validation set and achieved a similar AUC of 0.91 (sensitivity = 84%, specificity = 89%) using the same cut-off as in the training set, demonstrating its consistency and robustness (Fig. 4A, B). Covariant analysis also showed that the PDACatch classifier was independent of age, sex, tumour location and size (Fig. 4C–F).

Genes associated with the 56 markers of this classifier were annotated and a number of cancer-related genes or gene families were identified, including HOX family [35] and TBX family [35, 36] members (Additional file 5: Table S3). Several have been proposed for the detection of PDAC or other cancers in blood, including *BCAN*, *IKZF1*, *TBX15*, *BNC1* and *SHOX2* [17, 21, 32, 37, 38]. Gene Ontology (GO) analyses showed significantly enriched molecular function categories for DNA binding or transcription factor activity (Fig. 3E). It may be worth exploring whether these transcription factors have regulatory roles in PDAC carcinogenesis.

While testing CP samples, we found that the PDACatch classifier showed limited accuracy in stratifying CP from PDAC. We then rebuilt an SVM-based classifier to separate PDAC from CP plasma, which achieved an AUC of 0.85 for samples in the validation set (Additional file 2: Fig. S4A). This PDAC-CP classifier exhibited a consistent accuracy for PDAC across all stages (Additional file 2: Fig. S4B) with no significant covariate differences (Additional file 2: Fig. S4C-F). Although the limited CP samples likely have reduced performance during validation, the results did suggest potentially great feasibility in differentiating PDAC from CP using ctDNA methylation as markers to reduce misdiagnosis due to the lack of discriminatory symptoms.

### Comparison of the PDACatch classifier with serum CA19-9 levels

As mentioned earlier, serum CA19-9 is commonly used as a blood marker to stratify PDAC risk. Thus, it is necessary to compare the performance of the PDACatch classifier with CA19-9 in all samples with available test results for CA19-9 to assess PDACatch’s clinical utility and significance.

**Fig. 5:**
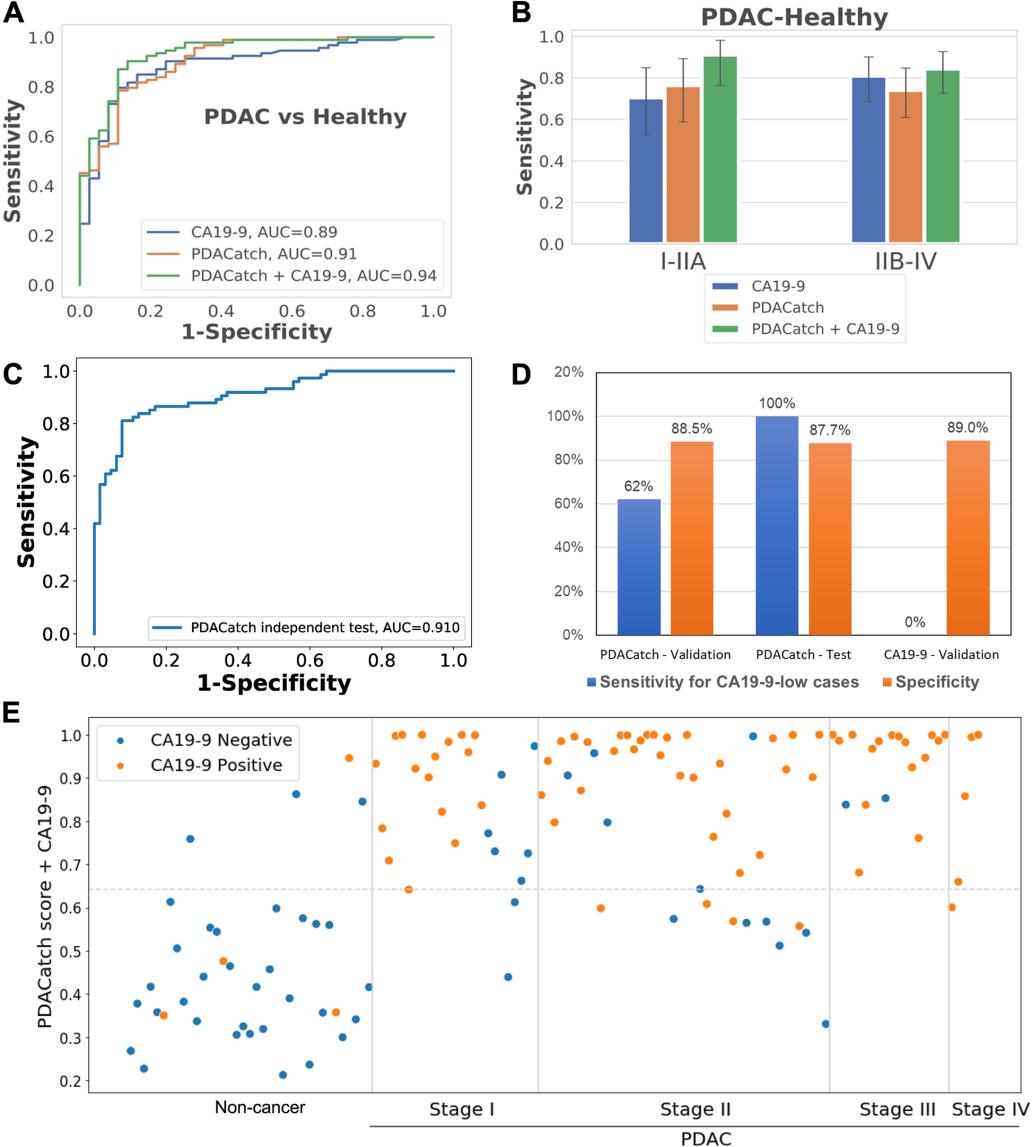
Independent test of the PDACatch classifier and its comparison and integration with CA19-9. **A** ROC curves of CA19-9, PDACatch and the combined classifier in differentiating PDAC and healthy controls in the training and validation sets. **B** Comparison of the sensitivities of CA19-9, PDACatch and the combinatorial classifier in classifying PDAC of different stages. The specificity was fixed at 89%. Error bars: 95% CI of sensitivity. **C** ROC curve of the PDACatch classifier on the independent test samples. **D** Comparison of the sensitivity of PDACatch and CA19-9 in detecting CA19-9-negative PDAC cases in the study cohorts. Note that the specificity was of the entire cohort by the classifier. **E** Predicted probability scores for noncancerous cases (*n* = 37) and Stage I (*n* = 25), Stage II (*n* = 44), Stage III (*n* = 18) and Stage IV (*n* =5). The same samples were also tested for CA19-9 levels. Orange dots show the CA19-9-positive cases (>37 U/ml), and blue dots show CA19-9-negative cases (≤37 U/ml)

We compared the classification accuracy by PDACatch and CA19-9 on all 92 PDAC and 37 healthy cases with known CA19-9 levels from the training and validation samples of Phase III (Fig. 1 and Table 2) and found that on balance, PDACatch was modestly more accurate than CA19-9, as demonstrated by the fact that PDACatch has a higher, or at least an equal, AUC score than CA19-9 for PDAC of each stage (Fig. 5A and Table 3). Importantly, PDACatch was more sensitive in detecting Stage I (sensitivity = 80 and 68% for PDACatch and CA19-9, respectively, Additional file 2: Fig. S5) or early-stage (I-IIa) PDAC plasma than CA19-9 (sensitivity = 76 and 70% for PDACatch and CA19-9, respectively). Note that in this comparison, PDACatch and CA19-9 had the same specificity of 89%. These results indicate that PDACatch may be more advantageous in detecting early PDAC cases than CA19-9.

**Table 3:** Performance of PDACatch, CA 19-9 and the combined model to classify all cases of the training and validation sets that have CA19-9 levels (bootstrapped 1000 repetitions at 95% CIs)

	PDACatch		CA19-9		Combined: PDACatch + CA19-9		
**PDAC**	**AUC (CI)**	**Sensitivity (CI)**	**AUC (CI)**	**Sensitivity (CI)**	**AUC (CI)**	**Sensitivity (CI)**	***p*value for AUC**
**I:*N*=25**	0.92 (0.85–0.97)	0.8 (0.59–0.93)	0.85 (0.75–0.93)	0.68 (0.46–0.85)	0.93 (0.88–0.98)	0.92 (0.74–0.99)	1.61E−06
**II:*N*=44**	0.88 (0.81–0.93)	0.68 (0.52–0.81)	0.88 (0.81–0.94)	0.75 (0.6–0.87)	0.92 (0.87–0.96)	0.77 (0.62–0.89)	5.42E−05
**I/IIA:*N*=34**	0.91 (0.84–0.96)	0.76 (0.59–0.89)	0.86 (0.78–0.93)	0.71 (0.53–0.85)	0.92 (0.86–0.97)	0.88 (0.73–0.97)	2.61E−05
**I/II:*N*=69**	0.89 (0.83–0.94)	0.72 (0.6–0.83)	0.87 (0.8–0.92)	0.72 (0.6–0.83)	0.93 (0.88–0.97)	0.83 (0.72–0.91)	1.59E−07
**III/IV:*N*= 23**	0.94 (0.89–0.98)	0.87 (0.66–0.97)	0.94 (0.88–0.99)	0.91 (0.72–0.99)	0.97 (0.93–0.99)	0.96 (0.78–1)	6.02E−06
**All stages:*N*=92**^**a**^	0.91 (0.85–0.95)	0.76 (0.66–0.84)	0.89 (0.83–0.94)	0.77 (0.67–0.85)	0.94 (0.89–0.97)	0.86 (0.77–0.92)	1.00E−07
		**Specificity (CI)**		**Specificity (CI)**		**Specificity (CI)**	
**Healthy:*N*=37**		0.89 (0.75–0.97)		0.89 (0.75–0.97)		0.89 (0.75–0.97)	

*p* values were calculated by the DeLong test

*PDAC* pancreatic ductal adenocarcinoma, *CA19-9* carbohydrate antigen 19-9, *CI* confidence interval

^a^Two PDAC cases had no stage information

Additionally, because 5~10% of PDAC cases do not have elevated CA19-9 levels due to genetic background [39], we specifically evaluated whether PDACatch can accurately detect PDAC cases considered to be negative in the CA19-9 test (termed CA19-9-negative cases, defined as CA19-9 levels lower than 37 U/ml, *N* = 21) from healthy controls (*N* = 33). Indeed, it correctly identified 13 out of 21 CA19-9-negative PDAC cases in the training and validation cohorts (sensitivity =54, 75 and 62% for the training, validation and combined cohorts, respectively) at a specificity of 91% (Fig. 5D and Additional file 2: Fig. S4A). Taken together, PDACatch not only outperformed CA19-9 in detecting early-stage PDAC patients but also accurately identified CA19-9-negative PDAC cases.

Lastly, we explored integrating CA19-9 with the PDACatch classifier to potentially maximize the model accuracy. To this end, we used cases from the training and validation sets for PDACatch that had known CA19-9 levels for the combinatorial model’s training (23 healthy, 62 PDAC) and validation (14 healthy, 31 PDAC), respectively. The combinatory classifier was trained by logistic regression and achieved an AUC of 0.93 (sensitivity = 82%, specificity = 87%); in validation, it achieved an AUC score of 0.96 (sensitivity = 94%, specificity = 93%), which was higher than either parental classifier (0.87 and 0.90 for CA19-9 and PDACatch, respectively) in classifying the same validation samples (Fig. 5, Table 3).

Because the combinatorial classifier had consistent performances in both training and validation cohorts, we further compared the combinatorial classifier’s performances with CA19-9 in classifying all the cases of these 2 cohorts. Indeed, the combinatorial classifier had an AUC of 0.94, higher than CA19-9 (AUC = 0.89, Fig. 5A–E and Table 3). Additionally, it was more sensitive than CA19-9 when classifying Stage I (sensitivity = 92 and 68% for the combinatorial classifier and CA19-9, respectively, *p* < 0.05, McNemar’s test, Additional file 2: Fig. S5) or early-stage PDAC plasma (I-IIa) (sensitivity = 88 and 70% for the combinatorial classifier and CA19-9, respectively, *p* < 0.05, McNemar’s test, Additional file 2: Fig. S6). These results suggest that early detection of PDAC may be improved by combining PDACatch and CA19-9.

### Independent test of the PDACatch classifier to distinguish PDAC and healthy plasma samples

To independently verify the PDACatch classifier’s utility in classifying PDAC plasma, we conducted a single-blinded classification on a cohort of preoperative PDAC (*N* = 74) plasma samples and healthy controls (*N* = 65, Fig. 1 and Table 1) obtained from ProteoGenex, a biobank in the USA. The PDACatch assay was performed on these samples, and the same classifier and cut-off were applied to label these samples as PDAC or normal.

The results showed that for the blind-test cohort, PDACatch achieved an AUC of 0.91 (sensitivity = 82%, specificity = 88%, Fig. 5C) in classifying PDAC cases, reaching a degree of accuracy that was essentially identical to that of the validation cohort (AUC = 0.91, sensitivity = 84%, specificity = 89%), further confirming its robustness and consistency. Stagewise, PDACatch detected early-stage PDAC (I-IIa) at a sensitivity of 80% and advanced-stage PDAC (IIb and above) at 83%, both of which were also consistent with the results of the validation cohort.

Importantly, PDACatch correctly identified all 7 CA19-9-negative PDAC samples in this cohort, achieving a sensitivity of 100% (Fig. 5D). While the number of such cases was relatively small in this cohort (22 of the 74 PDAC samples had serum CA19-9 levels measured), combined with the results of the same test on CA19-9-negative cases of the training and validation cohorts, it nonetheless demonstrated the PDACatch classifier’s consistent accuracy at detecting CA19-9-negative PDAC cases. Taken together, we found that the PDACatch classifier performed consistently in classifying PDAC plasma in the independent blind-test cohort as it did in the training and validation cohorts, confirming its robustness and utility for the noninvasive detection of PDAC in blood.

## Discussion

In this study, we investigated PDAC and control tissues and plasma to sequentially discover, develop, validate and test ctDNA methylation signatures for early PDAC detection. Methylation haplotype-based analyses were performed in the marker discovery phase to improve the specificity and robustness. PDACatch, a highly sensitive targeted methylation sequencing assay, was developed to integrate the most discriminating markers for PDAC. The 56-marker PDACatch classifier was built and performed better than CA19-9 in detecting early-stage PDAC and CA19-9-negative cases at high specificity. Most importantly, the PDACatch classifier was confirmed in an independent test to accurately classify PDAC plasma from healthy controls.

Taken together, these results are another step forward to achieving accurate detection of early PDAC using blood specimens, which is arguably the most cost-effective approach to reducing the high mortality rate of PDAC [40]. Neither imaging modalities nor non-CA19-9 serum markers have sufficient efficacy to detect early-stage PDAC [41]. Recently, ctDNA methylation has shown great potential as a blood marker to detect PDAC in its early stages [17]. Our results supported this notion, as both PDACatch-based classifiers achieved a high degree of sensitivity for early-stage PDAC plasma (76~82%), which is comparable to a recent multicancer early screening study’s results that also used DNA methylation changes as blood markers for cancers [42]. We further demonstrated that methylation markers can be combined with CA19-9 to maximize the overall accuracy, especially for CA19-9-negative PDAC cases that lack Lewis antigens and/or have early-stage disease [43]. This finding is especially meaningful to improve the diagnostic and prognostic stratification of PDAC patients.

During de novo marker discovery, we analysed MHBs of tissues and plasma to identify candidate ctDNA markers for PDAC in addition to AMF. When the methylation haplotype was first introduced, only comethylation was quantified (i.e., by the MHL measurement) [19], which limits the number of identified methylation markers. In this study, to increase the size of potential PDAC plasma markers, we expanded the analysis to include co-unmethylation by the UMHL measurement. We also explored weighing the length of the haplotype with an exponent of 3 in the MHL3 and UMHL3 measurements instead of just 1 in MHL and UMH to utilize the density of CpG sites in marker discovery. Finally, we specifically analysed whether the fully methylated (MHFm) or unmethylated (MHFu) haplotype was differentially expressed between PDAC samples and their controls.

Admittedly, applying the new measurement did increase the complexity in marker selection and modeling. However, PDACatch performed with notably high accuracy and consistency in all study cohorts that included both Chinese and non-Chinese populations, proving that a model incorporating these new types of methylation haplotypes can be stable and robust for populations of diverse genetic and epigenetic backgrounds, in addition to having more potential markers to choose from for model development. Practically, because the new measurements such as UMHL were all developed using a principle or formula similar to that of MHL, they can be easily implemented by researchers who are reasonably well-versed with MHL.

In addition to diagnostic, monitoring and prognostic applications [44, 45], ctDNA methylation has been investigated in recent years as a possible early detection biomarker in PDAC [17, 46–49]. Most of these studies were based on relatively small sample sizes and evaluated individual differentially methylated CpG sites or genes, which were typically selected from literature searches or by in silico prediction and might not be able to capture the complex biology of PDAC. In the present study, we conducted a comprehensive discovery and validation process in four sequential phases: (1) biomarker discovery; (2) assay development; (3) training, validation and testing; and (4) assay integration. Our classifier was trained and validated in a relatively large Chinese cohort and tested in an independent dataset of Caucasian individuals. Early-stage and CA19-9-negative cases, two important target groups for the early detection of PDAC, were analysed separately. Moreover, MHBs rather than individual CpGs were analysed for marker discovery, during which novel metrics quantifying the methylation status of MHBs were used to obtain the most representative measurement to identify differentially methylated MHBs.

Several limitations should be acknowledged. Our preliminary results from using methylation markers to differentiate PDAC plasma from CP were encouraging. However, they need to be further validated using a larger number of CP samples. The sample sizes of several key categories of cases, namely, Stage I and CA19-9-negative PDAC, were also limited (Additional file 2: Fig. S2). In addition to the high cost and complexity, ctDNA tests likely suffer from the same problems of insufficient sensitivity and specificity as traditional biomarkers when applied to population screening and early cancer diagnosis, given that the fraction of ctDNA was extremely small in total plasma DNA in early-stage tumours [50]. Although our test demonstrated improved accuracy using PDACatch over CA19-9 for early-stage PDAC, its sensitivity and specificity are still not good enough for early detection or screening purposes in PDAC and need further improvement. Last, a considerable number of PDAC patients with advanced-stage disease were included in our cohorts, which might overestimate PDACatch’s sensitivity. The effectiveness of the PDACatch assay in early PDAC detection needs further evaluation in a large multicentre prospective study.

## Conclusions

In summary, we conducted a de novo genome-wide screening using methylation haplotype-based analyses for PDAC-specific DNA methylation markers, and built a PDACatch classifier for early PDAC detection. Despite the limitations mentioned above, we believe that our study is an important step forward in reaching the goal of accurately noninvasively detecting early-stage PDAC to reduce the high mortality rate of PDAC. It will not only benefit early PDAC detection, but its methodology and analyses may play the foundation to develop DNA methylation-based diagnostics for other cancers.

## Supplementary Information

**Additional file 1.** Supplementary methods.

**Additional file 2: Figure S1.** Sample size of tissue samples (A) and plasma sample (B) in Phase I, Phase II and Phase III. The reused samples were showed in dot. **Figure S2.** Sample sizes of the key categories. Stage I (A) and CA19-9-negative PDAC (B) were shown. **Figure S3.** Overview of incremental feature selection of PDAC-specific MHB to build PandaX classifiers. **Figure S4.** Performance of PDAC-CP classifier in differentiating PDAC from CP plasma and covariate analysis. A. ROC of PDACatch classifying PDAC and CP plasma. B. PDACatch scores across different type of samples. In panel B, samples were labelled with cohorts (Train/Validation), pathological types (H: Healthy; CP: chronic pancreatitis) and clinical stages of PDAC (I, II, I-IIA and IIB-IV). For C-F, PDAC-CP classifier’s scores of PDAC and CP plasma samples were grouped by gender (A), age (B), tumour location (C) and tumour size (D). Wilcoxon rank sum test. ns: 0.05 < *p* <= 1.0; *: 1.00e-02 < *p* <= 5.00e-02; **: 1.00e-03 < *p* <= 1.00e-02; ***: 1.00e-04 < *p* <= 1.00e-03; ****: *p* <= 1.00e-04. **Figure S5.** Sensitivity by CA19-9, PDACatch and the combinatorial classifier (PDACatch + CA19-9) on Stage I PDAC plasma samples of Phase III samples. **Figure S6.** Performance of PDACatch- and PDAC-CP- classifiers in CA19-9-negative cases. Both PDACatch- (A) and PDAC-CP- (B) classifiers accurately detected CA19-9-negative PDAC cases.

**Additional file 3: Table S1.** Gene-set enrichment analysis (GSEA) of pathways (GO catergories) significantly represented in PDAC hypermethylated or PDAC hypomethylated MHB associated genes.

**Additional file 4: Table S2.** PDAC markers selected from genome-wide DNA methylation profiling experiments significantly enriched in the marker sets curated form literatures.

**Additional file 5: Table S3.** Marker lists and annotations of PandaX models.

## Data Availability

All data, analytic methods and study materials will be made available to other researchers upon request via emailing to liangzy@pumch.cn.

## Abbreviations

AMF: Average methylation fraction
AUC: Area under the curve
CA19-9: Carbohydrate antigen 19-9
cfDNA: Cell-free DNA
CHNMU: Changhai Hospital, Navy Medical University
CHNMU: Changhai Hospital, Navy Medical University
CP: Chronic pancreatitis
ctDNA: Circulating tumour DNA
EUS-FNA: Endoscopic ultrasound-guided fine needle aspiration
GO: Gene Ontology
GSEA: Gene set enrichment analysis
MHB: Methylation haplotype blocks
MHFm: Fully methylated haplotype fraction
MHFu: Fully unmethylated haplotype fraction)
MHL: Methylation haplotype load
PDAC: Pancreatic ductal adenocarcinoma
PUMCH: Peking Union Medical College Hospital
ROC: Receiver operating characteristic
RRBS: Reduced representation bisulfite sequencing
SVM: Support vector machine
UMHL: Unmethylation haplotype load
