# Supplementary material for: Noninvasive detection of pancreatic ductal adenocarcinoma using the methylation signature of circulating tumour DNA
Source: BMC Med. 2022 Nov 25;20:458. doi: 10.1186/s12916-022-02647-z (PMC9701032; doi:10.1186/s12916-022-02647-z)
Supplement: Supplementary file 1 — Additional file 1. Supplementary methods. [file 12916_2022_2647_MOESM1_ESM.docx]

**Additional File 1: Supplementary methods**

## Differentially methylated region selection from RRBS data

MHBs overlapping with repeat regions were discarded. MHB-level metrics (AMF, MHL, MHL3, UMHL and UMHL3) were calculated for the MHBs that have sequencing coverage of 10-fold or more. Samples with fewer than 25000 covered MHBs were removed from analyses. MHBs detected in over 90% samples were kept to build MHB matrices.

PDAC-specific MHBs were discovered by tissue-vs-tissue(T2T) and tissue-vs-plasma(T2P) comparisons. In either comparison, MHB profiles of tissue or plasma samples were subsampled without replacement for 125 times, followed by a 4-fold cross validation. In each cross validation, Wilcoxon rank-sum test was used to identify differentially methylated MHBs (FDR<0.05). A logistic regression model was built to predict the remainder test samples. If AUC of test dataset >= 0.75, the marker set was recorded. Finally, markers with a repeat occurrence of over 300 were selected.

GREAT(Genomic Regions Enrichment of Annotations Tool) with default parameters was used to analyze genes associated with candidate markers. We also collected 819 genes that have been reported as methylation markers for PDAC detection from literatures.

## Sequencing data preprocessing

First, Illumina paired-end sequencing reads were merged to single reads by pear(0.9.6) with parameter “-j 4 -v 20 -t 30 -n 30” to recover high quality original DNA fragments. Adapters at the end of reads were trimmed by trim_galore(0.6.0). The preprocessed reads were mapped to CT and GA converted HG19 by bismark(v0.17.0) with bowtie2(V2.3.1) and parameter(num_threads=4, seed_length=32, seed_mismatch=1). For target sequencing reads, unique molecular identifiers(UMIs) were extracted from each read after trimming. After mapping, UMI joined unique methylation haplotypes were extracted from BAM files with in-house Python scripts.

## Methylation haplotype measurements

Candidate methylation haplotype blocks (MHBs) were constructed as described previously. The CpG sites within each MHB tend to be tightly co-regulated on the epigenetic status at the level of single DNA molecules. We evaluated multiple block-level quantitative metrics in order to identify the most informative measurement for each target region. Such metrics included AMF (average methylation fraction), MHL(methylation haplotype load), UMHL(unmethylation haplotype load), MHFm(fully methylated haplotype fraction) and MHFu(fully unmethylated haplotype fraction).

**AMF:** AMF was defined as the average methylation level for all CpG sites in a specific target region. All detected CpG alleles divided by all methylated CpG alleles of target region

$$\frac{\sum_{i}^{M} N_{C,i}}{\sum_{i}^{M} \left( N_{C,i}+N_{T,i} \right)}$$

Where *i* represents a CpG site in this target region, M is the total number of CpG sites in this target region, N_T, i_ represents the number of thymines observed at CpG site *i*, N_C,_*_I_* represents the number of cytosines observed at CpG site *i*.

**MHL, MHL3, UMHL and UMHL3:** MHL was defined as in Guo et. al., which is the normalized fraction of methyl­ated haplotypes at different lengths.

$$MHL=\frac{\sum_{i=1}^{l} w_{i}\times P\left( {MH}_{i} \right)}{\sum_{i=1}^{l} w_{i}}$$

$l$ is the length of haplotypes, and $P({MH}_{i})$ is the fraction of fully succes­sive methylated CpGs within $i$ loci. $w_{i}$is the weight for $i$-locus haplotype. Options for weights are $w_{i}=i$ for MHL and $w_{i}=i^{3}$ for MHL3. Similar to MHL and MHL3, UMHL and UMHL3 are normalized fraction of unmethyl­ated haplotypes at different lengths.

**MHFm and MHFu:** The fully methylated haplotype fraction (MHFm) metric was computed for each fully methylated haplotype over each targeted region using the equation:

$${MHFm}_{i,h}=\frac{N_{i,h}}{N_{i}}$$

Where *i* is the current locus, *h* is the current haplotype, *N_i,h_* is the number of reads at the current locus containing the current haplotype, and *Ni* is the total number of reads covering the current locus. MHFu is the fraction of fully unmethylated haplotypes.

## Function and pathway analyses

Both GO enrichment and GSEA analyses were implemented by R package clusterProfiler (v3.16.1). Hypergeometric test was used identify enriched GO categories. p-values were adjusted by the Benjamini-Hochberg procedure.

## Statistical analysis

Statistical analyses were performed in R 3.5.0. Chi-square tests were utilized to test the difference of methylation level distribution between case and control groups (10 bins with equal intervals). Measurements with the smallest chi-squared test p-value of each target were selected as the methylation status of the corresponding target regions. Binomial confidence intervals for sensitivity and specificity were calculated using the Clopper-Pearson method. To assess whether the difference observed between AUCs from the CA19-9 and PandaX and the CA19-9 alone models was statistically significantly different from 0, we considered a test statistic T (T = AUC_CA19-9_ − AUC_CA19-9+PandaX_)2/ (*s*^2^ _CA19-9_ + *s*^2^ _CA19-9+PandaX_), which looks at the difference in AUC between the two models divided by the sum of the variances from the two models. The fact that this test statistic followed a χ^2^ distribution with 1 degree of freedom under the null hypothesis was used to calculate a resulting *P* value. A bootstrap percentile confidence interval (CI) approach was used to estimate a 95% CI for the AUC (1000 times).
