# Supplementary material for: Noninvasive detection of pancreatic ductal adenocarcinoma using the methylation signature of circulating tumour DNA
Source: BMC Med. 2022 Nov 25;20:458. doi: 10.1186/s12916-022-02647-z (PMC9701032; doi:10.1186/s12916-022-02647-z)
Supplement: Supplementary file 2 — Additional file 2: Figure S1. Sample size of tissue samples (A) and plasma sample (B) in Phase I, Phase II and Phase III. The reused samples were showed in dot. Figure S2. Sample sizes of the key categories. Stage I (A) and CA19-9-negative PDAC (B) were shown. Figure S3. Overview of incremental feature selection of PDAC-specific MHB to build PandaX classifiers. Figure S4. Performance of PDAC-CP classifier in differentiating PDAC from CP plasma and covariate analysis. A. ROC of PDACatch classifying PDAC and CP plasma. B. PDACatch scores across different type of samples. In panel B, samples were labelled with cohorts (Train/Validation), pathological types (H: Healthy; CP: chronic pancreatitis) and clinical stages of PDAC (I, II, I-IIA and IIB-IV). For C-F, PDAC-CP classifier’s scores of PDAC and CP plasma samples were grouped by gender (A), age (B), tumour location (C) and tumour size (D). Wilcoxon rank sum test. ns: 0.05 < p <= 1.0; *: 1.00e-02 < p <= 5.00e-02; **: 1.00e-03 < p <= 1.00e-02; ***: 1.00e-04 < p <= 1.00e-03; ****: p <= 1.00e-04. Figure S5. Sensitivity by CA19-9, PDACatch and the combinatorial classifier (PDACatch + CA19-9) on Stage I PDAC plasma samples of Phase III samples. Figure S6. Performance of PDACatch- and PDAC-CP- classifiers in CA19-9-negative cases. Both PDACatch- (A) and PDAC-CP- (B) classifiers accurately detected CA19-9-negative PDAC cases. [file 12916_2022_2647_MOESM2_ESM.docx]

**
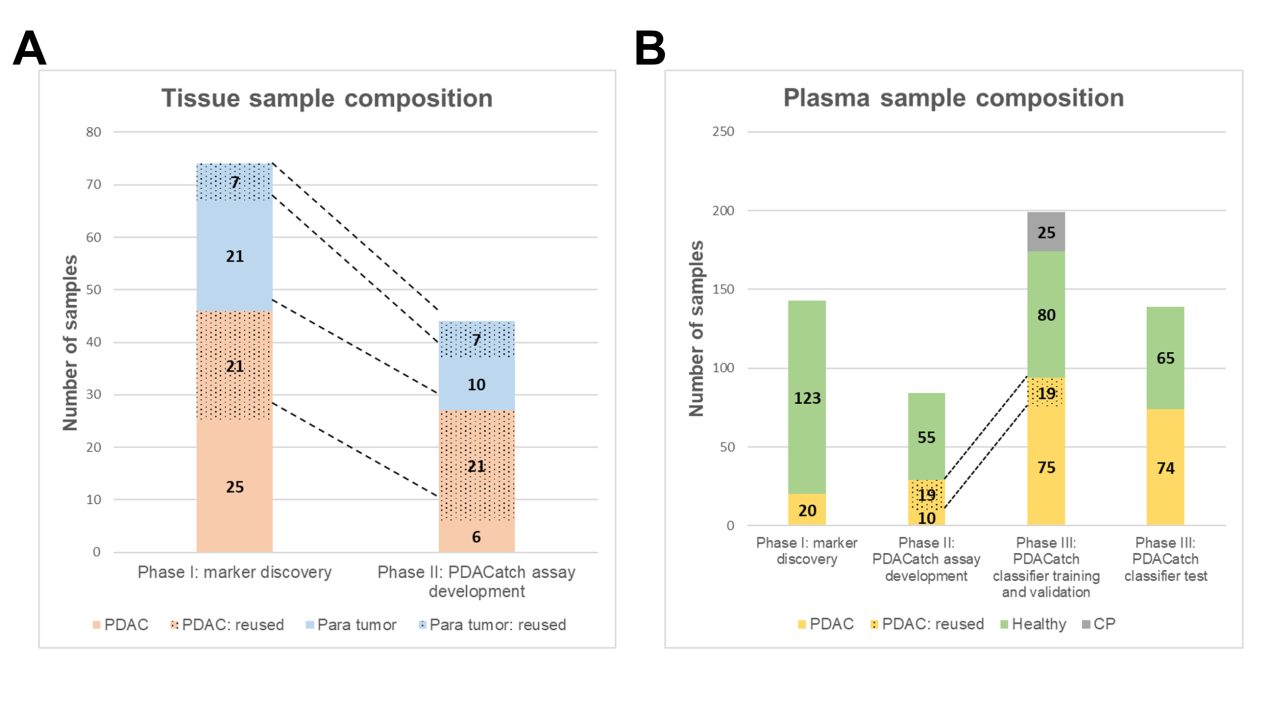
**

**Additional File 2: Figure S1** Sample size of tissue samples (A) and plasma sample (B) in Phase I, Phase II and Phase III. The reused samples were showed in dot.


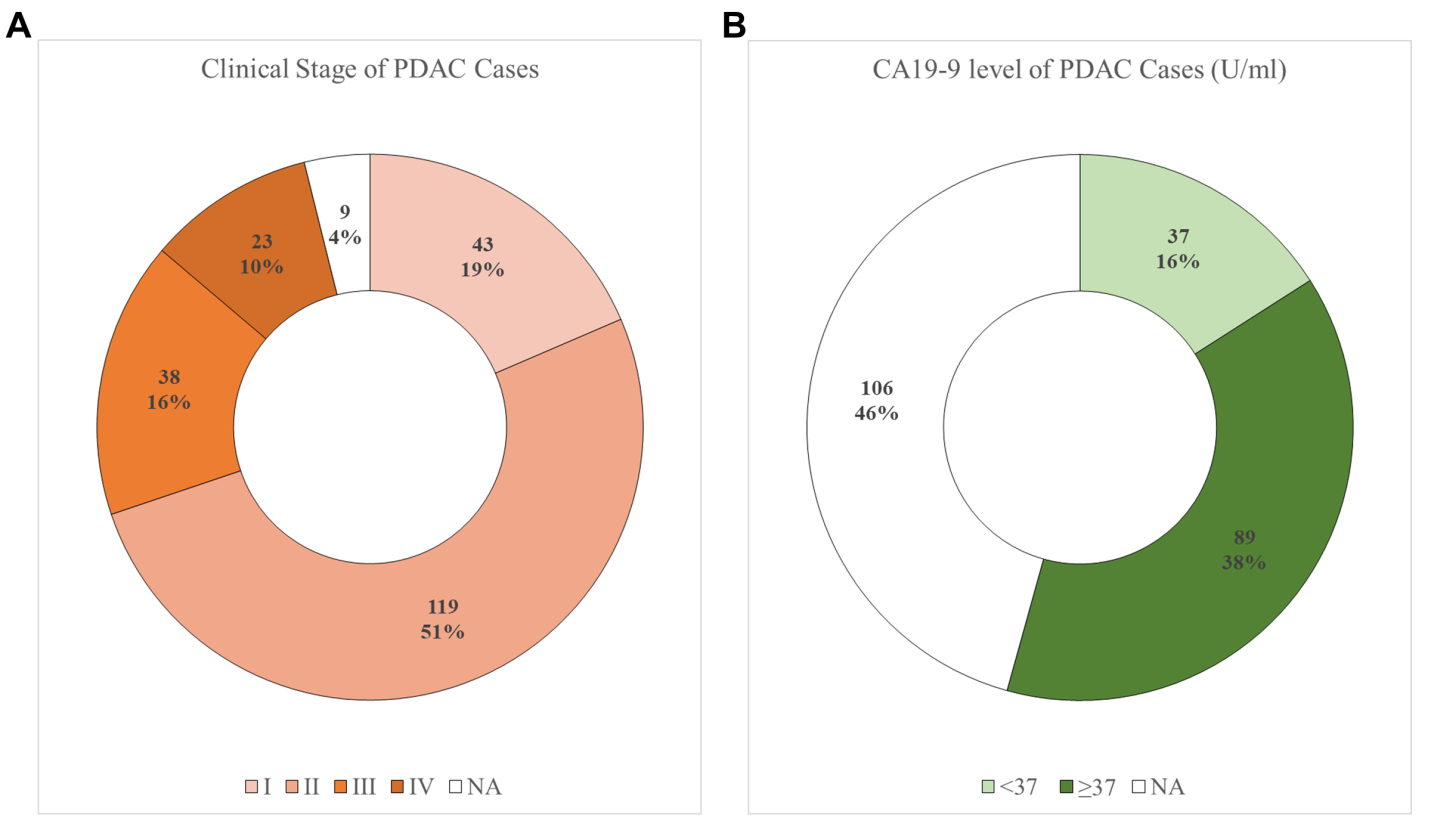


**Additional File 2: Figure S2** Sample sizes of the key categories: Stage I (A) and CA19-9-negative PDAC (B) were shown.

**
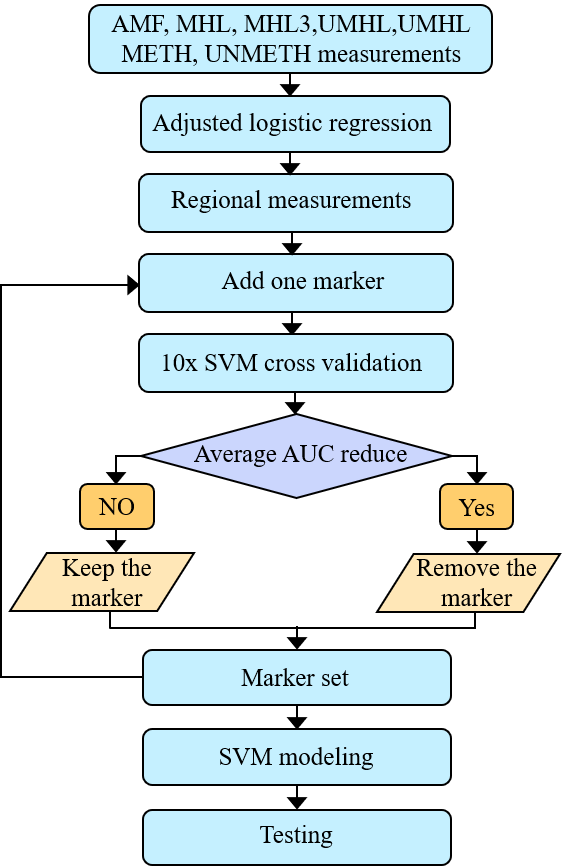
**

**Additional File 2: Figure S3** Overview of incremental feature selection of PDAC-specific MHB to build PandaX classifiers.

**
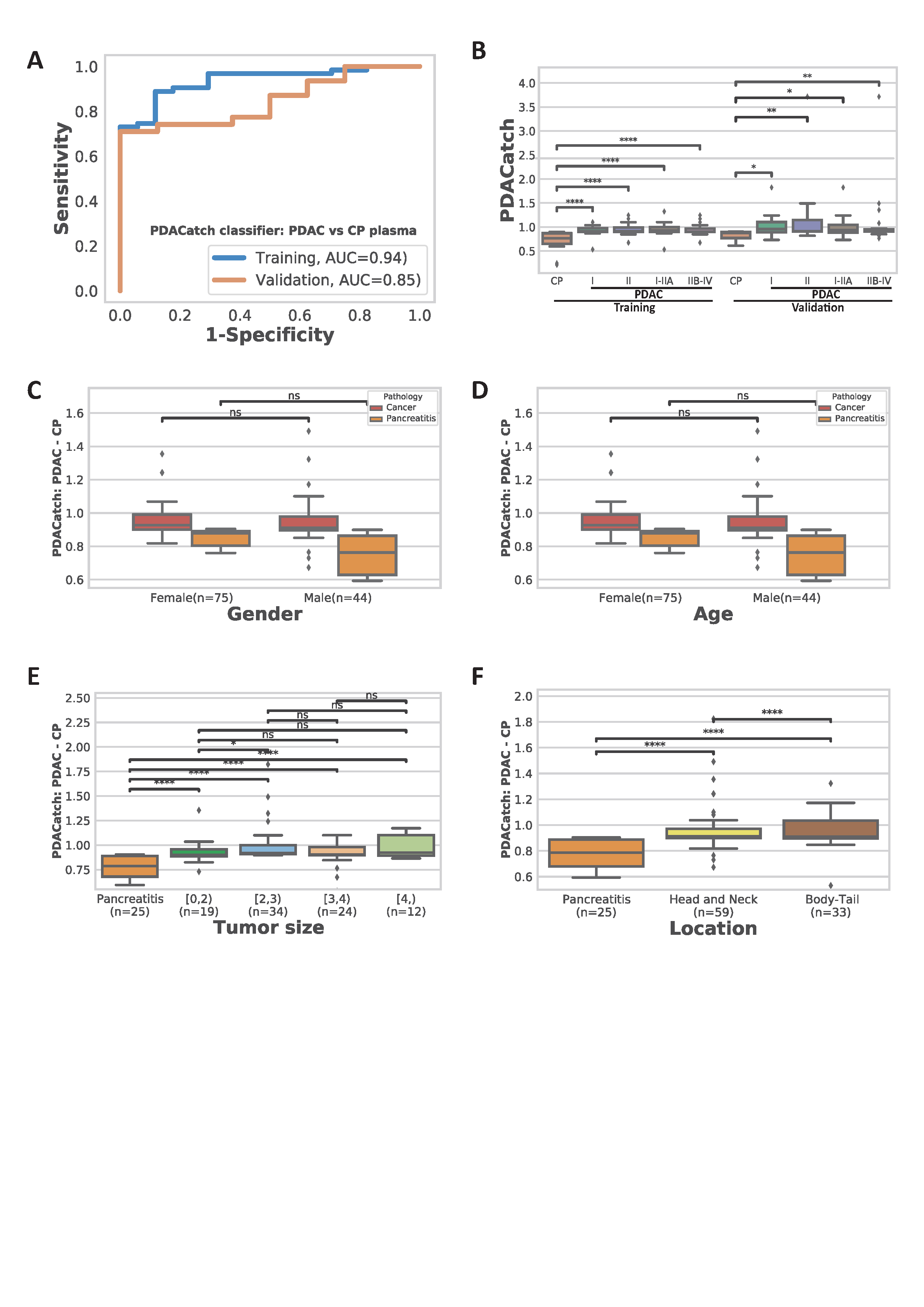
**

**Additional File 2: Figure S4** Performance of PDAC-CP classifier in differentiating PDAC from CP plasma and covariate analysis. A. ROC of PDACatch classifying PDAC and CP plasma. B. PDACatch scores across different type of samples. In panel B, samples were labeled with cohorts (Train/Validation), pathological types (H: Healthy; CP: chronic pancreatitis), and clinical stages of PDAC (I, II, I-IIA and IIB-IV). For C-F, PDAC-CP classifier’s scores of PDAC and CP plasma samples were grouped by gender (A), age (B), tumor location (C) and tumor size (D). Wilcoxon rank sum test. ns: 0.05 < p <= 1.0; *: 1.00e-02 < p <= 5.00e-02; **: 1.00e-03 < p <= 1.00e-02; ***: 1.00e-04 < p <= 1.00e-03; ****: p <= 1.00e-04.

**
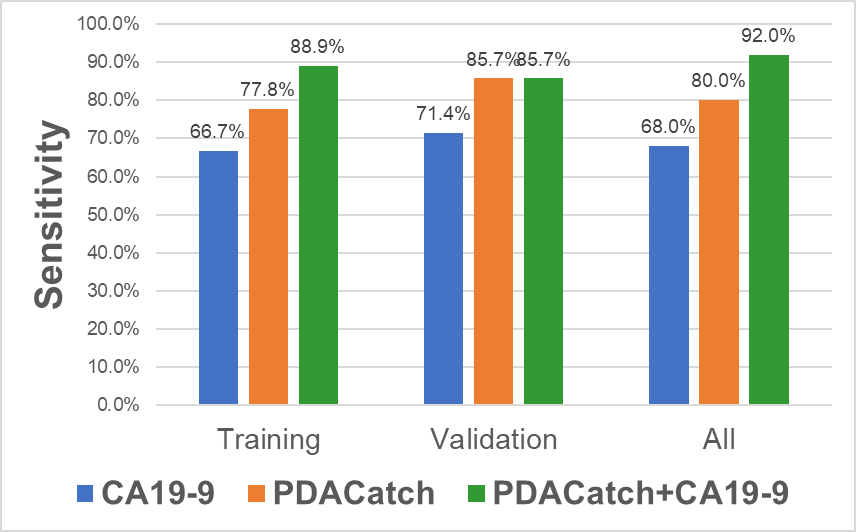
**

**Additional File 2: Figure S5** Sensitivity by CA19-9, PDACatch and the combinatorial classifier (PDACatch + CA19-9) on Stage I PDAC plasma samples of Phase III samples.

**
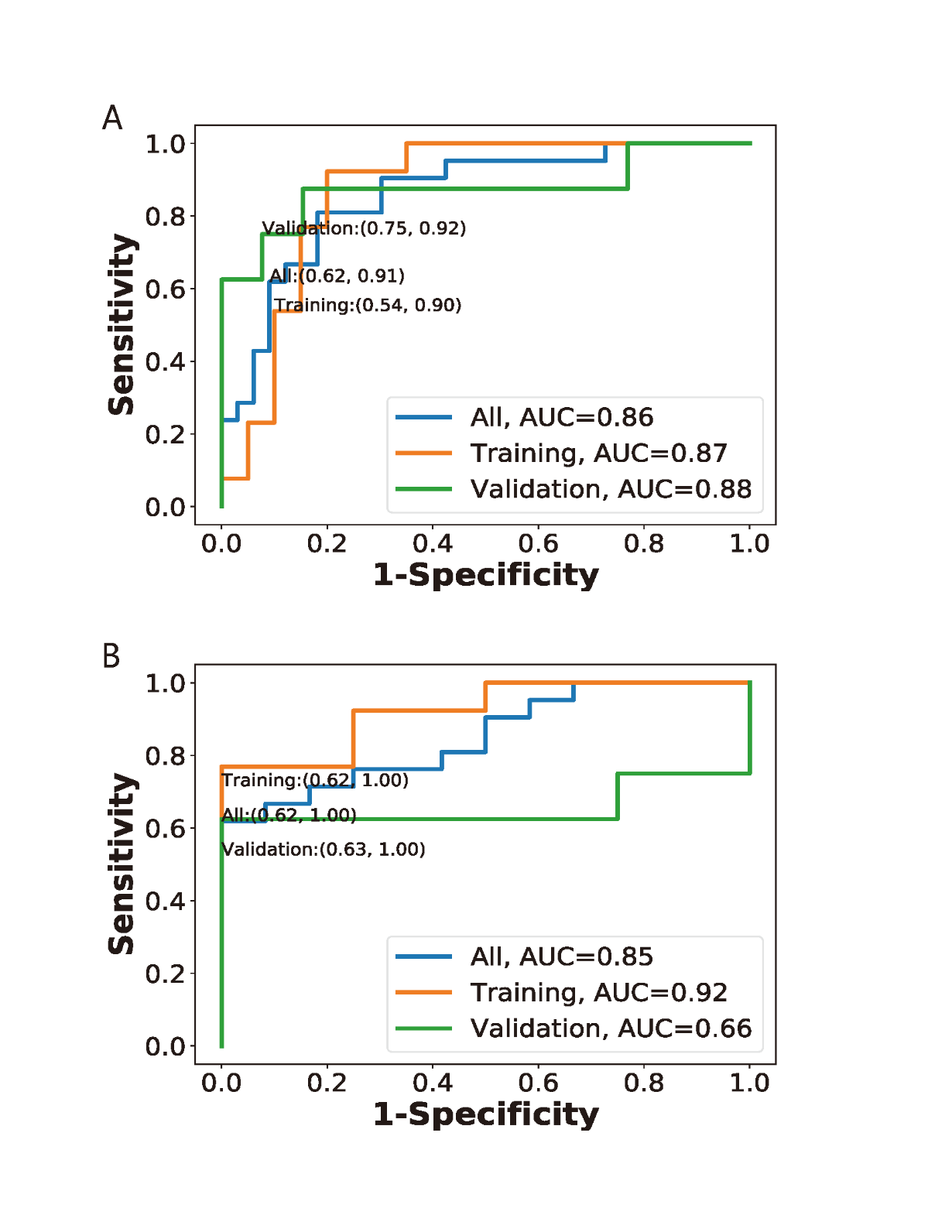
**

**Additional File 2: Figure S6** Both PDACatch- (**A**) and PDAC-CP- (**B**) classifiers accurately detected CA19-9-negative PDAC cases.
